# Online Sustained-Attention Testing as an Adjunct Outcome Measure in Personalized Spine Surgery Research: A Study Protocol and Methodological Framework Using the 90-Second CVAT

**DOI:** 10.3390/jpm16100490

**Published:** 2026-09-23

**Authors:** Kai-Uwe Lewandrowski, Jülide Elmali, Elisa Hauschild, Luisa Terschek, Magdalena Tommasi, Kristen Michelle Watson, Benedikt Burkhardt, Joachim Oertel, Alexander Paul Leopold Lewandrowski, Thomas Lundquist, Rossano Kepler Alvim Fiorelli, Tobias Fritz, Emmanouil Liodakis, Sergio Luis Schmidt

**Affiliations:** 1 Division of Personalized Pain Research and Education, Center for Advanced Spine Care of Southern Arizona, Tucson, AZ 85712, USA; 2 Department of Orthopedic Surgery, University of Arizona Tucson Campus, Tucson, AZ 85721, USA; 3 Department of Orthopaedics, Fundación Universitaria Sanitas, Bogotá 110131, Colombia; 4 Department of Orthopedics, Hospital Universitário Gaffrée e Guinle, Universidade Federal do Estado do Rio de Janeiro, Rio de Janeiro 20270-004, Brazil; 5 Medical Faculty, Saarland University, 66421 Homburg, Germany; juel00001@teams.uni-saarland.de (J.E.); krwa00001@teams.uni-saarland.de (K.M.W.); 6 Wirbelsäulenzentrum Zürich/Spine Center WSC, Hirslanden Klinik Zürich, 8032 Zurich, Switzerland; 7 Department of Neurosurgery, Saarland University Medical Center and Saarland University Faculty of Medicine, 66421 Homburg, Germany; joachim.oertel@uks.eu; 8 Department of Biological Sciences, Dornsife College of Letters, Arts & Sciences, University of Southern California, Los Angeles, CA 90089, USA; 9 William Beaumont School of Medicine, Oakland University, Rochester, MI 48309, USA; 10 Department of General and Specialized Surgery, Hospital Universitário Gaffrée e Guinle, Universidade Federal do Estado do Rio de Janeiro, Rio de Janeiro 20270-004, Brazil; fiorellirossano@hotmail.com; 11 Klinik für Unfall-, Hand- und Wiederherstellungschirurgie, Saarland University Medical Center, 66421 Homburg, Germany; tobias.fritz@uks.eu (T.F.); emmanouil.liodakis@uks.eu (E.L.); 12 Neurology Post-Graduate Program, Hospital Universitário Gaffrée e Guinle, Universidade Federal do Estado do Rio de Janeiro, Rio de Janeiro 20270-004, Brazil

**Keywords:** spine surgery, personalized medicine, Continuous Visual Attention Test, CVAT, online testing, sustained attention, reaction-time variability, digital performance indicator, telehealth, responder analysis

## Abstract

**Background/Objectives:** This protocol article presents a methodological framework and does not itself report a new validation dataset. Personalized spine surgery increasingly requires outcome measures that capture recovery domains beyond pain, disability, imaging, neurological status, complications, and revision surgery. Sustained-attention instability may reflect pain-related cognitive burden and may influence rehabilitation adherence, work capacity, medication management, and postoperative self-care. Building on published CVAT evidence and additional unpublished findings from a completed independent online known-groups cohort, this protocol article specifies a standardized framework for integrating the online 90 s Continuous Visual Attention Test (CVAT) into personalized spine surgery research. **Methods:** The proposed protocol combines conventional clinical-outcome assessment, patient-reported outcome measures, and repeated online CVAT testing at baseline and follow-up. Its primary implementation is a prospective, longitudinal observational cohort using a harmonized common core; disease-specific modules prospectively specify the diagnosis, procedure, clinical endpoint, sample size, and analysis. Prospective studies should define baseline as the first protocol-specified CVAT assessment, preferably before surgery or another index intervention. Retrospective and registry-based cohorts may support cross-sectional analyses or prospective trajectories beginning at digital enrollment when no preintervention CVAT measurement is available. Candidate spine cohorts include lumbar disk herniation, cervical disk herniation/radiculopathy, cervical myelopathy, lumbar spinal stenosis, and expanded postoperative or revision cohorts. The primary CVAT performance endpoint is variability of reaction time (VRT); VRT/RT is a key secondary and sensitivity endpoint, while mean reaction time (RT), omission errors, and commission errors are secondary endpoints. **Protocol Components:** Existing peer-reviewed evidence supports the brief CVAT paradigm, chronic-spine-pain known-groups discrimination, and remote administration; additional completed online data independently reproduced the principal VRT and VRT/RT pattern in chronic spine pain. The protocol specifies eligibility criteria, task parameters, scoring and preprocessing, remote-testing procedures, tokenized access, device and input-method documentation, session-validity checks, feasibility outcomes, exploratory responder and non-responder definitions, longitudinal assessment schedules, sample-size examples, missing-data and multiplicity procedures, and a staged pathway from established foundations to postoperative prognostic and clinical-utility evaluation. **Conclusions:** The online 90 s CVAT has an empirical foundation as a brief, remotely deployable performance-based measure and is proposed as an adjunct—not a replacement for PROMs, examination, imaging, or clinical judgment. Prospective spine surgery validation is required to establish postoperative reliability, responsiveness, meaningful-change thresholds, incremental prognostic value, and clinical utility; paired technical studies remain necessary when strict cross-platform interchangeability is claimed.

## 1. Introduction

Personalized medicine is reshaping spine surgery by encouraging a shift from standardized treatment algorithms toward individualized diagnosis, surgical planning, risk stratification, rehabilitation, and outcome monitoring. In modern spine care, personalization includes more than anatomical localization and procedure selection. It increasingly incorporates pain mechanisms, frailty, psychological health, sleep, medication exposure, rehabilitation capacity, work demands, imaging phenotype, biologic status, and patient-specific recovery goals.

The Continuous Visual Attention Test (CVAT) is a computerized Go/No-Go paradigm designed to assess attentional performance using measures including reaction time (RT), variability of reaction time (VRT), omission errors (OE), and commission errors (CE). A 90 s version was developed to improve clinical feasibility, and prior work demonstrated that sustained attention can be measured using this brief computerized task, with VRT showing agreement between the 15 min and 90 s versions [1]. In chronic spine pain, a 90 s CVAT study identified VRT and VRT/RT as candidate markers of sustained-attention instability [2]. A peer-reviewed study subsequently compared online and face-to-face administration of the CVAT, providing a published basis for proposed remote testing while leaving device- and population-specific equivalence to be established [3].

Traditional spine surgery outcome assessment remains centered on pain scores, disability indices, health-related quality-of-life instruments, neurological examination, imaging, complication rates, revision surgery, and patient satisfaction. These measures are indispensable. Common examples include the visual analog scale (VAS) and numeric rating scale (NRS), Oswestry Disability Index (ODI) [4], Neck Disability Index (NDI) [5], modified Japanese Orthopaedic Association (mJOA) score [6,7], Zurich Claudication Questionnaire [8], EQ-5D, SF-36, and PROMIS instruments. However, these instruments do not directly measure whether a patient can sustain attention, maintain psychomotor consistency, or perform cognitive tasks reliably under chronic nociceptive load. PROMs report the patient experience, whereas CVAT samples performance under standardized task demands.

Chronic pain is associated with impaired attention, executive dysfunction, working-memory deficits, and altered cognitive performance [9,10,11]. These effects may be clinically relevant in spine patients because sustained attention contributes to rehabilitation adherence, driving safety, work performance, fall avoidance, medication management, and postoperative self-care.

The available effect sizes are informative but not directly transferable to postoperative prediction. In the published laptop study of 84 adults with chronic lumbar or cervical pain and 118 pain-free controls, adjusted group effects were large for VRT (partial η^2^ = 0.44) and VRT/RT (partial η^2^ = 0.39) [2]. A separate completed tokenized online known-groups study included 401 participants, comprising 271 adults with chronic spinal pain and 130 pain-free controls. Because this study has not yet been published, its findings are reported here as unpublished study data (authors’ data on file). The chronic-pain group had higher age- and sex-adjusted VRT (adjusted difference 17.077 ms, 95% CI 7.725–26.429; *p* < 0.001; partial η^2^ = 0.031) and VRT/RT (adjusted difference 0.039, 95% CI 0.014–0.063; *p* = 0.002; partial η^2^ = 0.023). Both differences persisted in the prespecified sensitivity sample after the exclusion of anticipatory responders. The online effects were smaller than in the published laptop cohort but reproduced the principal outcome pattern. Together, these findings support prospective evaluation of CVAT as an adjunct candidate measure; they do not establish causality, postoperative responsiveness, or routing utility.

The transition from in-office laptop administration to remote online testing is clinically important but methodologically nontrivial. Because the CVAT is reaction-time-based, online implementation must consider device variability, keyboard or touchscreen input, browser behavior, home-environment distractions, and potential timing artifacts. Therefore, remote CVAT testing has been framed as a standardized digital outcome tool with predefined quality safeguards, not simply as an informal online questionnaire. In peer-reviewed modality work, 130 participants contributed between-group data and 50 completed counterbalanced within-person testing; mean performance did not differ by modality, while VRT agreement and test–retest reliability were moderate (ICC = 0.53 and 0.54, respectively) [3]. The independent online cohort provides additional direct known-groups evidence in chronic spine pain. Because that cohort used an independent sample rather than paired cross-mode testing and did not retain complete device, browser, and software-build metadata, it demonstrates replication of the clinical signal rather than strict numerical interchangeability. Remaining technical work therefore concerns test–retest measurement error and claims of device- or mode-specific equivalence; it is not a prerequisite for prospective research using a standardized tokenized online implementation.

The purpose of this paper is to propose a methodological framework for using the online 90 s CVAT as an adjunct outcome measure in personalized spine surgery research. The proposed workflow is summarized in the Graphical Abstract. The CVAT is not presented as a replacement for PROMs, imaging, neurological examination, or clinical judgment. Instead, it is proposed as a brief, objective, performance-based measure that may help characterize sustained-attention instability and support future responder/non-responder phenotyping in spine surgery cohorts. The novelty is therefore methodological rather than a new disease-association result: prior publications evaluated task duration, administration modality, or CVAT associations in clinical samples [1,2,3], whereas this article provides the first spine-focused common-core protocol linking a fixed task and scoring algorithm to diagnosis-specific PROMs, repeated surgical follow-up, remote quality controls, motor and contextual covariates, a prioritized statistical plan, and staged validation before any routing decision. This reusable protocol is needed so future disease-specific spine studies can be implemented and compared consistently.

## 2. Conceptual Rationale

### 2.1. Why Attention Matters in Spine Surgery Recovery

Spine surgery outcomes are multidimensional. Two patients may undergo the same operation for the same diagnosis yet have different recovery trajectories. One patient may experience pain relief, improved function, reduced medication use, and rapid return to work. Another may have persistent pain, sleep disturbance, fear avoidance, reduced stamina, delayed rehabilitation, or incomplete functional recovery despite technically successful decompression or fusion.

Attention and cognitive consistency may influence these trajectories. Patients with chronic pain frequently describe mental fatigue, slowed processing, poor concentration, and difficulty maintaining attention. These symptoms may not be fully captured by pain scores or disability indices. A brief objective test of attentional stability may therefore add a clinically relevant dimension to postoperative assessment. These clinical consequences remain hypotheses in spine surgery; the protocol is designed to test them rather than presume them.

The 90 s Go/No-Go paradigm is not process-pure. VRT can reflect attention consistency, but performance may also be affected by psychomotor speed, response inhibition, visual processing, instruction comprehension, fatigue, motivation, sleep, mood, acute pain, medication exposure, digital familiarity, and motor variability. Construct validation will therefore include a recognized attention comparator and discriminant measures of mood and motor speed/dexterity. In cervical myelopathy, dominant-hand function must be quantified and modeled because disease-related hand impairment may affect response latency independently of attention.

### 2.2. Why VRT and VRT/RT Are Central

Mean RT may reflect alertness, psychomotor speed, age, fatigue, medication effects, or motor limitations. VRT, by contrast, captures trial-to-trial inconsistency and may better reflect sustained-attention instability. VRT/RT further normalizes variability relative to mean response speed, helping distinguish attentional instability from generalized slowing [1,2].

For the common-core scoring algorithm, mean RT is the arithmetic mean of valid correct-target latencies, and VRT is their within-person sample standard deviation: VRT = sqrt[Σ(RT_i_ − mean RT)^2^/(n − 1)]. VRT/RT is mathematically the dimensionless intraindividual coefficient of variation. Because a quotient can inherit dependence on its denominator and non-normality [12], VRT is the primary performance endpoint; VRT/RT is a key secondary/sensitivity endpoint, and a complementary model will analyze VRT with mean RT entered separately as a covariate.

For personalized spine surgery research, VRT is the primary CVAT performance endpoint. VRT/RT is a key secondary and sensitivity endpoint. RT, OE, and CE remain useful secondary outcomes but may be more sensitive to psychomotor speed, task comprehension, response inhibition, or measurement context. CE is based on only 18 non-target opportunities; OE and CE will be analyzed as counts/proportions and checked for floor or ceiling effects rather than treated as precise continuous biomarkers.

### 2.3. Why Online Testing Is Important

In-person testing limits scalability. Spine surgery follow-up increasingly includes telehealth, hybrid care, remote PROM collection, decentralized trials, and long-term outcomes registries. An online CVAT can be administered using individualized secure links before clinic visits, during postoperative follow-up, or at predefined research time points. Figure 1 illustrates how the online CVAT can be integrated into a personalized digital spine-outcomes pathway. Until predefined equivalence limits are met, online and in-office data and keyboard- and touchscreen-based data should not be pooled uncritically.

Remote deployment may be particularly useful for long-term follow-up. Patients who are at least two years postoperative can be re-contacted, complete PROMs remotely, and undergo current online CVAT assessment without requiring travel. Without a preoperative or earlier CVAT score, however, such participants contribute cross-sectional or retrospective–prospective association data and cannot support within-person claims about recovery-related change. These adaptations must therefore be analyzed separately from the prospective longitudinal core.

## 3. Common-Core Study Protocol and Research Framework

### 3.1. Overview

The proposed framework integrates three major outcome domains: subjective symptom burden, clinical and functional recovery, and objective attentional performance measured using the online 90 s CVAT. This multidimensional model is intended to support personalized recovery phenotyping rather than replace existing outcome measures. The primary implementation is a prospective, longitudinal observational cohort enrolling consecutive eligible adults before surgery or another prespecified spine intervention and repeating the same CVAT, patient-reported outcome measure (PROM), and clinical assessment battery at follow-up. Each disease-specific module prospectively specifies its participating sites, recruitment window, diagnosis and procedure, primary hypothesis and clinical outcome, target sample size, and ethics and registration details.

The common core may also support retrospective cohorts with prospective online follow-up, cross-sectional long-term assessments, registry-based digital outcome studies, and telehealth workflows. These are secondary design adaptations with distinct estimands, potential biases, and sample-size requirements. They should not be pooled with prospective within-person change analyses without design-specific justification. All modules share the task parameters, scoring definitions, contextual metadata, session-quality rules, CVAT outcome hierarchy, and minimum analysis standards specified below.

The framework builds on an existing empirical evidence base rather than an untested platform. Peer-reviewed studies have established the feasibility of the 90 s paradigm, demonstrated discrimination between patients with chronic spine pain and pain-free controls, and supported online administration [1,2,3]. The principal VRT and VRT/RT pattern was also reproduced in the independent online cohort described above.

The remaining objectives are ordered to prevent premature clinical interpretation: (1) module-specific technical integrity and feasibility; (2) test–retest reliability, practice effects, measurement error, and strict administration-mode or device agreement when interchangeability is claimed; (3) convergent and discriminant construct validity and reference-data development; (4) longitudinal responsiveness; (5) incremental prognostic value beyond established outcomes; and (6) clinical utility of any proposed CVAT-informed routing rule. Later clinical stages should proceed only after the relevant measurement requirements have been satisfied (Figure 2).

### 3.2. Online CVAT Task, Scoring, Preprocessing, and Remote-Testing Safeguards

The online 90 s CVAT is a computerized Go/No-Go visual-attention task in which participants respond to target stimuli and withhold responses to non-target stimuli. The standardized sequence begins with a 10 s error-free practice followed by 90 scored trials: 72 targets (80%) and 18 non-targets (20%). Each stimulus is displayed for 250 ms followed by a 750 ms blank interval, yielding a 1000 ms stimulus-onset asynchrony. One repeat practice is permitted after an error; failure of both practice attempts ends the session and triggers staff review rather than generation of an analyzable score. The scored task yields RT, VRT, OE, and CE; VRT/RT is calculated as a normalized variability measure [1,2,3].

For trial *i*, latency is measured locally in milliseconds from the stimulus-onset event to the first qualifying response event. A valid correct-target RT is 100 to <1000 ms. Responses <100 ms are flagged as anticipatory; absence of a valid target response within the trial epoch is classified as an OE; and any response during a non-target trial is classified as a CE. Practice trials, non-target trials, omitted targets, anticipatory responses, repeated keydown/autorepeat events, and responses outside the trial epoch are excluded from RT and VRT calculations. Mean RT, VRT, and VRT/RT are then calculated as defined in Section 2.2, and the same scoring algorithm is applied across devices.

A scored session requires completion of all 90 trials and at least 37 valid correct-target RTs, consistent with the published requirement of more than half of the 72 target trials [1]. Prespecified invalidity flags include OE ≥ 50%; CE = 18 with OE = 0, suggesting a response to every stimulus; or an unresolved technical interruption. RT or VRT values more than 3 sample standard deviations from the study distribution are flagged for review rather than automatically deleted. The primary scoring algorithm performs no winsorization or transformation; robust, transformed, and outlier-exclusion analyses are reported only as sensitivity analyses.

Participants receive an individualized, study-linked token with a predefined expiry window (Figure 3). Tokenization limits uncontrolled retesting and links a session to a pseudonymous study identifier, but it does not prove participant identity, prevent link sharing, guarantee completion, or eliminate practice effects at later visits. Identity must therefore be verified through the approved study portal, a second authentication factor, or staff-confirmed release. One replacement token may be issued only after a documented technical failure; the original attempt and failure reason remain in the audit trail, and a second valid attempt is not substituted merely because the first score is unfavorable.

Reaction-time measurement uses client-side monotonic, high-resolution timestamps for stimulus onset and response. Server-receipt time is retained for provenance and transmission monitoring but is not used for RT calculation. Each record stores the task/scoring version, browser and operating system, device class, input method, screen dimensions, reported refresh rate when available, timing deviations or dropped-frame indicators, focus/visibility changes, reloads, network interruption, practice attempts, and trial-level events. Every software build undergoes version-specific timing checks before clinical correlation data are pooled.

Participants should be instructed to complete the test in a quiet environment, use a desktop or laptop computer with a physical keyboard whenever possible, avoid multitasking and external distractions, sit comfortably, complete the practice trial, and continue the task even after making an error. If tablet or phone administration is used, the input method should be documented because touchscreen response times may differ from keyboard-based response times. Follow-up testing should use the same device, browser family, input method, response hand, and approximately the same time of day as baseline whenever feasible.

Before each session, the protocol records sleep duration and quality, current pain, analgesic or sedative use and time since the last dose, caffeine intake within 4 h, alcohol or recreational drug exposure within 24 h, acute illness, distraction, and assistance. The standardized practice is repeated at every visit; assessment sequence and prior exposure count are modeled to estimate learning effects.

These safeguards define the minimum operational quality-control framework for remote CVAT administration (Table 1). Additional requirements for reproducible implementation, including standardized participant instructions, minimum trial- and session-level data capture, and prospective study-module registration, are specified in Section 3.3.

### 3.3. Minimum Requirements for Reproducible Implementation

To facilitate consistent implementation across sites and disease-specific modules, the common-core protocol defines minimum requirements for participant instructions, auditable trial- and session-level data capture, and prospective study-module registration (Figure 4). These requirements are intended to preserve reproducibility across sites, software builds, and future disease-specific applications while allowing prespecified module-specific adaptations.

#### 3.3.1. Standard Participant Instructions

The approved language version of the standardized participant instruction should state:

“You will see a star or a diamond in the center of the screen. Using your dominant hand, press the spacebar or approved response surface as quickly as possible when the star appears. Do not respond when the diamond appears. Keep looking at the center, continue even if you make an error, and do not switch tabs or applications.”

The participant completes a 10 s error-free practice before beginning the scored task. One repeat practice is permitted after an error. Staff may clarify the instructions before the scored task begins but may not coach responses during the test.

Validated translations or protocol-approved language versions should preserve the same task instructions and response rules. Any language-specific adaptation should be documented prospectively within the corresponding study module.

#### 3.3.2. Minimum Trial-Level and Session-Level Data

The auditable trial-level file should contain, at minimum, the pseudonymous study ID, planned assessment time point, token ID, task/scoring version, local stimulus-onset timestamp, stimulus class, response timestamp or timestamps, computed latency, qualifying-response flag, OE/CE status, focus/visibility state, and any timing deviation.

The session-level file should contain, at minimum, the number of practice attempts, session start and end time and time zone, completion status, valid-trial count, RT, VRT, VRT/RT, OE and CE counts and proportions, device/browser/operating-system/input/screen metadata, interruptions, contextual variables, replacement-token linkage when applicable, final validity status, invalidity reason, and reviewer/date information.

Raw trial-level data should be retained in accordance with the approved data-retention plan so that summary scores can be reproduced after software updates, algorithm verification, or prespecified reanalysis. Derived scores should remain traceable to the task/scoring version used to generate them.

#### 3.3.3. Minimum Study-Module Registration

Before recruitment begins, each disease-specific module should prospectively record its study design and setting; participating sites; target diagnosis and procedure; recruitment strategy; primary hypothesis and estimand; primary clinical outcome and assessment time point; target sample size and sample-size calculation; inclusion and exclusion criteria; required comparator, motor, and contextual measures; device policy; technical-validation subset, when applicable; missing-data and multiplicity plan; ethics and registration identifiers; platform operator, host, and software version; and whether clinical staff are blinded to research CVAT results.

Each module should also identify any deviation from the common-core protocol prospectively and provide a scientific justification. Module-specific adaptations may include altered follow-up intervals, additional clinical outcomes, disease-specific motor assessments, language versions, or device restrictions, but the shared task parameters, scoring definitions, metadata requirements, and core validity rules should remain fixed unless a protocol amendment explicitly specifies otherwise.

## 4. Clinical Cohorts and Example Applications

The common-core protocol described in Section 3 is designed for pragmatic application across common spine surgery diagnoses. Figure 5 illustrates how the standardized online CVAT framework can support disease-specific study modules while preserving methodological consistency across the broader research program. Each module may be implemented independently or within a coordinated multicenter design and retains diagnosis- and procedure-specific outcomes.

### 4.1. Lumbar Disk Herniation

Lumbar disk herniation is a practical initial cohort because outcomes after discectomy, microdiscectomy, or endoscopic decompression are commonly assessed using leg-pain relief, back pain, ODI, recurrent herniation, analgesic use, and return to work. Potential questions include whether radicular pain relief correlates with improved VRT or VRT/RT, whether persistent neuropathic leg pain is associated with persistent attentional instability, and whether online CVAT metrics identify patients with delayed return to work despite improvement in leg pain. These are prespecified research questions; CVAT results do not determine treatment during validation.

### 4.2. Cervical Disk Herniation and Cervical Radiculopathy

Patients treated for cervical radiculopathy can be assessed using neck pain, arm pain, NDI, neurological examination, and satisfaction. Online CVAT testing may help determine whether persistent neck pain or radicular symptoms are associated with sustained-attention instability, and whether structural or radicular recovery is discordant with pain-related cognitive load. The proposed role is to test incremental information beyond symptoms and neurological assessment, not to diagnose radiculopathy or replace structural evaluation.

### 4.3. Cervical Myelopathy

Cervical myelopathy involves spinal cord dysfunction, gait impairment, hand dysfunction, balance problems, and variable neurological recovery. Although the CVAT is not a myelopathy test, it may provide a useful adjunctive cognitive-performance measure. Potential questions include whether CVAT parameters are associated with mJOA score, hand dexterity, gait impairment, balance, fall risk, and postoperative neurological recovery. Because the test requires a motor response, each myelopathy module must quantify dominant-hand function using the mJOA upper-extremity domain plus a prespecified dexterity measure. Motor impairment is modeled explicitly and evaluated in sensitivity analyses; inability to generate a stable response excludes the participant from RT/VRT clinical analyses but not from feasibility accounting.

### 4.4. Lumbar Spinal Stenosis

Lumbar spinal stenosis is commonly evaluated using walking tolerance, claudication symptoms, the ODI, the Zurich Claudication Questionnaire, quality of life, and satisfaction after decompression or decompression plus fusion [8]. Online CVAT testing may help determine whether improvements in walking and claudication parallel improvement in VRT or VRT/RT, and whether persistent attentional instability identifies patients with ongoing pain-related cognitive burden despite adequate decompression. Older age, medication burden, sleep, digital familiarity, and device access are particularly important feasibility and bias variables in this cohort.

### 4.5. Future Expanded Cohorts

After feasibility is established, this framework can be extended to adult spinal deformity, revision surgery, failed back surgery syndrome, sacroiliac joint pain, vertebral compression fracture, spinal infection, tumor surgery, neuromodulation, regenerative interventions, and patient-specific implant studies. Each extension requires a diagnosis-specific protocol amendment, outcome definition, sample-size calculation, and ethics determination; no CVAT threshold is inherited automatically from another cohort (Table 2).

## 5. Participants and Outcome Measures

### 5.1. Eligibility Criteria

Potential inclusion criteria include adults undergoing or having undergone spine surgery, a defined diagnosis such as lumbar disk herniation, cervical disk herniation, cervical myelopathy, lumbar spinal stenosis, ability to complete PROMs and online CVAT testing, adequate corrected vision, adequate motor ability to operate the selected response device, ability to provide informed consent, and access to a suitable device for online testing. Prospective modules recruit before intervention to obtain baseline CVAT; long-term first-contact cohorts are explicitly classified as cross-sectional adaptations.

Potential exclusion criteria include known dementia or severe cognitive impairment, neurological disease unrelated to the spine condition that substantially affects attention, active major psychiatric instability, active substance use disorder, uncorrected visual impairment, inability to understand instructions, severe upper-extremity motor impairment preventing valid response recording, acute postoperative delirium or unstable medical condition, or recent medication changes likely to substantially alter attentional performance. Cognitive screening uses a brief validated instrument (Mini-Cog by default for participants aged ≥65 years or whenever concern is present) [14]; a low score prompts clinician review rather than automatic exclusion unless consent or task comprehension is compromised. Psychiatric status is identified by structured medical-record review and PHQ-9/GAD-7 screening [15,16]; stable depression or anxiety is retained, measured, and adjusted for rather than broadly excluded.

Studies should document age, sex, education, pain duration, baseline pain intensity, medication exposure, sleep disturbance, depression, anxiety, work status, compensation or litigation status when ethically appropriate, surgical diagnosis, procedure type, complications, and revision surgery. These variables may affect CVAT performance and should be considered in multivariable analyses. Medication exposure is not treated as a simple binary exclusion. Each visit records drug name/class, dose, schedule, recent change, and time since the last analgesic, opioid, gabapentinoid, muscle relaxant, benzodiazepine, sedative-hypnotic, stimulant, antidepressant, or other attention-relevant medication. Stable prescribed regimens are retained and modeled; acute intoxication, unplanned rescue sedation, or a major recent change leads to rescheduling or a prespecified sensitivity exclusion. Screening logs also record eligible nonparticipation, device/access barriers, technical assistance, usability, burden, completion time, and technical failure to quantify digital exclusion [13].

### 5.2. CVAT and Clinical Outcomes

The primary CVAT performance endpoint is VRT. VRT/RT is a key secondary and sensitivity endpoint. Secondary CVAT endpoints include RT, OE, and CE. This hierarchy is based on published brief CVAT and clinical evidence [1,2,3] while acknowledging the denominator dependence of VRT/RT and the limited precision of CE. Table 3 distinguishes operational definitions and published construct associations from the authors’ spine-specific hypotheses.

Pain should be measured using the VAS or NRS, preferably separating axial pain from radicular pain. Diagnosis-specific disability instruments should include the ODI for lumbar disorders [4], the NDI for cervical disorders [5], mJOA for cervical myelopathy [6,7], and the Zurich Claudication Questionnaire for lumbar spinal stenosis [8]. General outcomes may include EQ-5D, SF-36, PROMIS Global Health, PROMIS Physical Function, PROMIS Pain Interference, return to work, walking distance, analgesic use, physical therapy completion, hand dexterity, gait function, balance, fall history, and need for additional interventions. A recognized attention comparator (for example, SART, a brief Psychomotor Vigilance Test, or an NIH Toolbox attention measure) is included in a nested construct-validation subset.

## 6. Responder and Non-Responder Definitions

PROM responders may be defined using prespecified clinically meaningful thresholds for pain, the ODI, the NDI, EQ-5D, SF-36, or PROMIS instruments. For lumbar disk herniation, responders may demonstrate meaningful leg-pain relief, ODI improvement, and return to function. For cervical radiculopathy, responders may demonstrate arm-pain relief, NDI improvement, and neurological improvement. For cervical myelopathy, responders may demonstrate mJOA improvement, stabilization of neurological decline, improved gait, or improved hand function. For lumbar spinal stenosis, responders may demonstrate improved walking tolerance, reduced claudication, ODI improvement, and improved quality of life.

Clinical responder status must be predefined within each disease module by naming the instrument, direction and magnitude of change, baseline-severity requirement, and follow-up time. Pain, disability, complications, revision, satisfaction, analgesic burden, and return to work should remain distinct outcomes unless a validated composite with a clear estimand is prospectively justified. A CVAT responder threshold is not currently validated for spine surgery. Continuous VRT trajectories are therefore primary; dichotomized CVAT response, normalization, minimum detectable change, and clinically important change are exploratory until spine-specific reliability and measurement error are estimated and thresholds are independently validated.

Figure 6 shows proposed PROM/CVAT phenotypes that may guide future validation studies. These phenotypes are not intended as stand-alone clinical triage rules; rather, they provide a structured basis for testing whether attentional performance adds incremental value to conventional outcomes. No decision threshold or clinical action is recommended by this protocol.

Available published CVAT studies do not constitute an online, spine-specific normative standard [1,2,3]. Establishing age-, sex-, education-, device-, and input-appropriate reference distributions is therefore an essential validation deliverable; until then, isolated “normalization” claims are exploratory.

## 7. Statistical Analysis Framework

### 7.1. Analysis Hierarchy and Primary Estimands

The common-core primary feasibility estimand is the proportion of initiated assessments yielding a valid scored session at each time point, with a 95% confidence interval and failure reasons. VRT is the primary performance variable. Each disease module prospectively designates one primary clinical estimand—for example, baseline-adjusted mean VRT change at 3 months or the time-by-clinical-responder interaction—and one primary clinical outcome. VRT/RT, RT, OE, CE, alternate time points, subgroup comparisons, and phenotype classifications are secondary or exploratory.

Baseline demographic, clinical, surgical, PROM, and CVAT variables should be summarized by diagnosis and procedure group. Continuous variables should be reported as means with standard deviations or medians with interquartile ranges depending on distribution. Categorical variables should be reported as frequencies and percentages.

CVAT variables may be compared between responders and non-responders using *t*-tests, Mann–Whitney U tests, ANOVA, ANCOVA, or non-parametric equivalents. Analyses should account for age, sex, education, pain duration, medication use, and baseline outcome severity. Prospective studies should use repeated-measures models or mixed-effects models to evaluate change in CVAT variables over time. These comparisons are secondary unless designated as the module’s single primary clinical estimand. Technical validation uses paired supervised and remote administrations and same-device test–retest assessment; VRT agreement is reported with absolute-agreement ICCs, 95% confidence intervals, and Bland–Altman bias and limits [17,18]. Prospective longitudinal analyses prioritize mixed-effects models with participant and site structure, baseline VRT, time, diagnosis/procedure, the prespecified clinical outcome, and time-by-outcome interaction. Device-by-time, motor impairment, practice sequence, and medication change are prespecified sensitivity effects.

Associations between CVAT variables and PROMs may be evaluated using Pearson or Spearman correlations. Regression models can test whether CVAT variables independently explain outcome variance after adjustment for confounders. Exploratory predictive models may test whether VRT or VRT/RT improves prediction of persistent pain, delayed rehabilitation, poor return to work, revision surgery, or prolonged postoperative care. Any prediction model should be developed and reported according to established guidance and externally validated before clinical use [19,20,21]. Observational studies should also follow relevant reporting principles such as STROBE [22]. A base model containing established clinical variables and PROMs is compared with a locked CVAT-augmented model. Prediction reporting includes calibration plots, calibration intercept and slope, discrimination, Brier score, bootstrap optimism correction, and external validation when available; AUC comparison alone is insufficient [20,21]. Decision-curve or net-benefit analysis is undertaken only after adequate calibration and discrimination.

Machine-learning analyses are not part of the common-core confirmatory plan. Any future machine-learning study requires a separate prospectively specified protocol, an adequately sized dataset and event count, locked features, internal optimism correction, and independent validation. CVAT data remain adjunctive and cannot replace clinician judgment. Table 4 summarizes the ordered analysis framework.

### 7.2. Sample-Size and Precision Framework

Because this is a reusable common-core protocol rather than one diagnosis-specific trial, a single sample size would be statistically inappropriate. Each activated module must register a design-specific calculation based on its primary estimand, anticipated attrition, clustering, and outcome prevalence. Published effects provide bounds rather than transferable assumptions: the chronic-spine-pain case–control study reported large cross-sectional VRT effects [2], but these cannot be used as an expected postoperative responder effect. Planning should therefore use conservative small effects unless credible target-module pilot data exist.

Worked two-sided examples at α = 0.05 and 80% power are 194 complete participants to detect r = 0.20 (243 enrolled with 20% attrition) and 199 complete paired observations to detect standardized within-person change dz = 0.20 (249 enrolled with 20% attrition). The same completed online cohort yielded 401 analyzable sessions, demonstrating practical deployment at scale, but its archived dataset lacked the complete approached, declined, incomplete, and technical-failure denominators needed to estimate a formal completion rate. Future modules must therefore prospectively define and report those denominators. As an illustrative design calculation, estimating an expected 80% valid-session rate with approximately ±5 percentage-point 95% precision requires about 246 initiated participants. The target for paired reliability modules should be calculated from the anticipated ICC, number of repeated measurements, and desired 95% confidence-interval precision rather than set by a universal fixed number. Prediction-module sample size is calculated from outcome prevalence, candidate parameters, expected model fit, and target shrinkage [20]. A proposed longitudinal schedule is shown in Table 5.

### 7.3. Missing Data, Multiplicity, and Robustness

Screening and analysis sets are defined prospectively: all eligible patients approached, all enrolled participants, all initiated CVAT sessions, all valid scored sessions, and the primary longitudinal analysis set. Missingness is tabulated by time, diagnosis, age, device access, and clinical outcome. Invalid CVAT sessions are not numerically imputed as observed scores; their reasons are feasibility outcomes. Mixed models use available repeated observations under a missing-at-random assumption. When covariate or clinical-outcome missingness is material, multiple imputation by chained equations includes exposures, outcomes, auxiliary variables, site, and missingness predictors [25]; complete-case and pattern-mixture or inverse-probability sensitivity analyses assess differential attrition. Each module has one primary clinical estimand tested at two-sided α = 0.05; prespecified secondary CVAT families use Holm adjustment or false-discovery-rate control [23,24]. Multicollinearity, nonlinearity, baseline adjustment, device-by-time interaction, medication changes, and cervical-myelopathy motor impairment are assessed prospectively.

## 8. Ethical, Regulatory, Data-Security, and Platform Considerations

The 90 s CVAT has been implemented in published clinical studies [1,2,3] and in the completed online cohort described above, which yielded 401 analyzable sessions. This demonstrates practical remote deployment at scale, although module-specific completion, invalid-session, technical-failure, usability, and burden rates must still be measured prospectively. In spine surgery research, CVAT is used as an adjunctive attention-related performance outcome and candidate prognostic measure alongside conventional clinical assessment.

Online CVAT data should be stored securely and handled according to institutional, national, and international data protection standards. The use of tokenized access, encrypted transmission, version control, and secure storage is recommended. Regulatory guidance for digital health technologies in clinical investigations supports careful attention to remote data acquisition, data integrity, and participant safety [26]. Minimum requirements include authenticated study access in addition to the token, pseudonymous identifiers, separation of identifiers from task data, TLS encryption in transit, encryption at rest, role-based least-privilege access, audit trails, controlled exports, tested backup/recovery, approved retention and deletion schedules, breach-response procedures, and documented data-controller/processor responsibilities under the applicable jurisdiction. The protocol and consent materials must identify hosting region, cross-border transfer, secondary-use and withdrawal/deletion limits, and who can view identifiable linkages.

The scientific CVAT paradigm and its research availability are described in prior publications [1,2,3]. The tokenized delivery layer has also undergone direct known-groups evaluation in that completed online cohort. In this protocol, the delivery layer is treated as an implementation mechanism and is not proposed for stand-alone diagnostic use. Every activated module must disclose the software developer, platform operator, host, data controller, task/scoring version, intellectual-property or licensing arrangement, testing fee (if any), and any author or institutional financial interest. Tokenization alone must not be described as proof of security or identity.

The online CVAT is intended as an adjunctive performance-based assessment of attentional stability and a candidate prognostic outcome in spine research. It is not proposed as a replacement for pain ratings, PROMs, neurological examination, imaging, or clinician judgment. Findings should be interpreted alongside clinical and contextual variables that may influence performance, including current pain intensity, sleep, medication exposure, mood, fatigue, neurological disease, motor function, device/input method, and testing conditions. These determinants should be recorded and evaluated as covariates or sensitivity factors; they do not negate the replicated association between chronic spine pain and increased reaction-time variability.

Remote testing introduces potential variability from device type, keyboard versus touchscreen input, browser performance, screen characteristics, environmental distractions, and participant behavior [27,28,29,30]. Nevertheless, published modality evidence [3] and the independent online cohort provide an empirical basis for remote CVAT research. The latter reproduced the principal chronic-spine-pain VRT and VRT/RT pattern but, because it used an independent cohort and lacked complete technical metadata, did not test strict numerical interchangeability with the published laptop cohort. Online studies should therefore use predefined quality checks, retain device/input/browser/software metadata, and prefer same-device longitudinal follow-up. Device or input changes are evaluated through device-by-time and same-device sensitivity analyses. Paired agreement or formal equivalence analyses are required before claiming interchangeability or pooling materially different modes without qualification; they are not a prerequisite for prospective association studies using a standardized internally consistent online protocol.

### Roadmap for Clinical Validation

Validation is cumulative rather than an all-or-none future requirement. Published work already supports the brief CVAT paradigm, chronic-spine-pain discrimination, and remote administration [1,2,3]; the completed online cohort additionally reproduced the expected known-groups differences in chronic spine pain. Stage 0 maintains software and timestamp integrity for each build. Stage 1 prospectively quantifies module-specific feasibility, usability, acceptability, digital exclusion, completion, and technical failure. Stage 2 estimates same-condition test–retest reliability, practice effects, measurement error, and formal supervised/remote or device/input agreement when interchangeability is intended. Stage 3 extends construct validation through recognized attention comparators, discriminant measures of motor speed/dexterity and mood, and population-appropriate reference distributions. Stage 4 evaluates responsiveness, minimum detectable change, and anchor-based clinically important change. Stage 5 evaluates incremental prognosis beyond PROMs and clinical factors with internal and external validation. Stage 6 tests whether a locked CVAT-informed routing rule improves decisions, outcomes, burden, harms, equity, or net benefit. Table 6 states the corresponding gates.

## 9. Discussion

This paper proposes a common-core protocol for integrating the online 90 s CVAT into personalized spine surgery research. The framework is intentionally pragmatic and reusable across prospective longitudinal observational cohorts and separately labeled retrospective–prospective, cross-sectional, telehealth, registry, and multicenter adaptations. Its task, scoring, metadata, quality-control, and analysis requirements provide a stable methodological foundation for future diagnosis-specific studies.

The central rationale is that spine surgery recovery is not fully captured by pain scores, disability indices, neurological examination, and imaging alone. Chronic spine pain and delayed postoperative recovery may involve sustained-attention instability, cognitive load, sleep disturbance, medication effects, and altered psychomotor consistency. The online CVAT provides a brief objective method for measuring one component of this broader recovery phenotype. CVAT is therefore evaluated alongside—not instead of—PROMs, examination, imaging, and conventional outcomes.

The proposed model is particularly relevant to personalized medicine. Patients with similar imaging findings and similar operations may follow different postoperative trajectories. Some may show concordant improvement in pain, disability, function, and attentional stability. Others may show discordant outcomes, such as pain improvement with persistent attentional instability, or persistent pain with normalized attention. These patterns may help refine follow-up decisions, rehabilitation intensity, medication review, sleep evaluation, and pain-management referral. At present these patterns are research phenotypes only. Personalization is operationalized as the future test of whether baseline or early VRT trajectory improves calibrated prediction of a prespecified outcome beyond diagnosis, procedure, pain, disability, sleep, mood, medication, motor function, and other established predictors. Only after external validation should an impact study determine whether CVAT-informed routing improves care compared with conventional information alone.

The strongest candidate CVAT performance marker is VRT, with VRT/RT retained as a key secondary and sensitivity measure. Prior work supports the 90 s CVAT as a brief measure of sustained-attention consistency [1], identifies large VRT and VRT/RT differences in chronic spine pain [2], and reports online/face-to-face and test–retest evidence [3]. The independent online cohort reproduced significantly higher age- and sex-adjusted VRT and VRT/RT in chronic spine pain, including after sensitivity exclusion of anticipatory responders. The convergent outcome pattern across the published laptop cohort and the larger online cohort supports prospective tokenized CVAT research in spine populations. Because the cohorts were independent, this replication supports online known-groups validity rather than paired numerical equivalence; it also does not establish postoperative responsiveness or incremental prognostic value.

Several limitations must be acknowledged. First, this is a framework paper and does not itself establish clinical utility. Second, online CVAT testing may be influenced by device and environment. Third, spine-specific thresholds for meaningful change in VRT or VRT/RT have not yet been established. Fourth, CVAT abnormalities do not identify a single mechanism. Fifth, responder/non-responder classification must be prospectively validated before it can guide clinical triage. The completed unpublished online dataset produced 401 analyzable sessions and therefore demonstrates practical deployment at scale, but complete screening, incomplete-session, technical-failure, and device/browser denominators were not retained; a formal completion rate and device-specific equivalence could not be estimated. Published modality agreement was moderate and was not established in an older postoperative spine population using every device/browser combination. The 90 s task is not process-pure, and motor function is a direct measurement determinant in cervical myelopathy. OE and especially CE may show floor or ceiling effects. Digital access may select younger or more advantaged participants. Thus, module-specific feasibility, reliability, and technical transportability remain prospective outcomes rather than reasons to characterize the online platform as wholly unvalidated.

Future studies should establish test–retest reliability in spine patients, define clinically meaningful-change thresholds, compare online and in-office administration in paired designs, evaluate preoperative-to-postoperative trajectories, and test whether CVAT-enhanced models improve prediction of persistent pain, return to work, rehabilitation failure, or unplanned escalation of care. Multicenter studies should also evaluate device effects, remote-testing adherence, and generalizability across languages, countries, and health systems. Association must be distinguished from prediction, and prediction from clinical utility: correlation with pain does not establish future recovery or demonstrate that changing attention changes outcome.

## 10. Conclusions

The online 90 s CVAT has an empirical foundation as a brief, remotely deployable performance-based measure and is proposed as an adjunct to conventional spine surgery outcome assessment [1,2,3]. Published laptop findings and the independent online cohort converge on VRT and VRT/RT as the most consistent chronic-spine-pain signals. By measuring trial-to-trial attention-related performance consistency through VRT, with VRT/RT, RT, OE, and CE as secondary or sensitivity outcomes, the CVAT may help characterize recovery phenotypes not fully captured by PROMs, imaging, or neurological examination alone. Conventional outcomes remain indispensable comparators and predictors.

This methodological framework supports future personalized spine surgery research by combining remote CVAT testing with pain, disability, clinical outcomes, and longitudinal responder analysis. If prospectively validated, online CVAT-based attentional phenotyping may contribute to more individualized postoperative monitoring, targeted rehabilitation, telehealth triage, and precision spine care. Routine clinical implementation and CVAT-informed patient routing are not recommended at this stage. They depend on postoperative demonstration of test–retest measurement error, responsiveness, meaningful-change thresholds, incremental calibrated prediction, external validation, and clinical net benefit. Additional paired device/input studies are required when strict cross-platform interchangeability is claimed, while module-specific technical monitoring remains part of every prospective implementation.

## Figures and Tables

**Figure 1:**
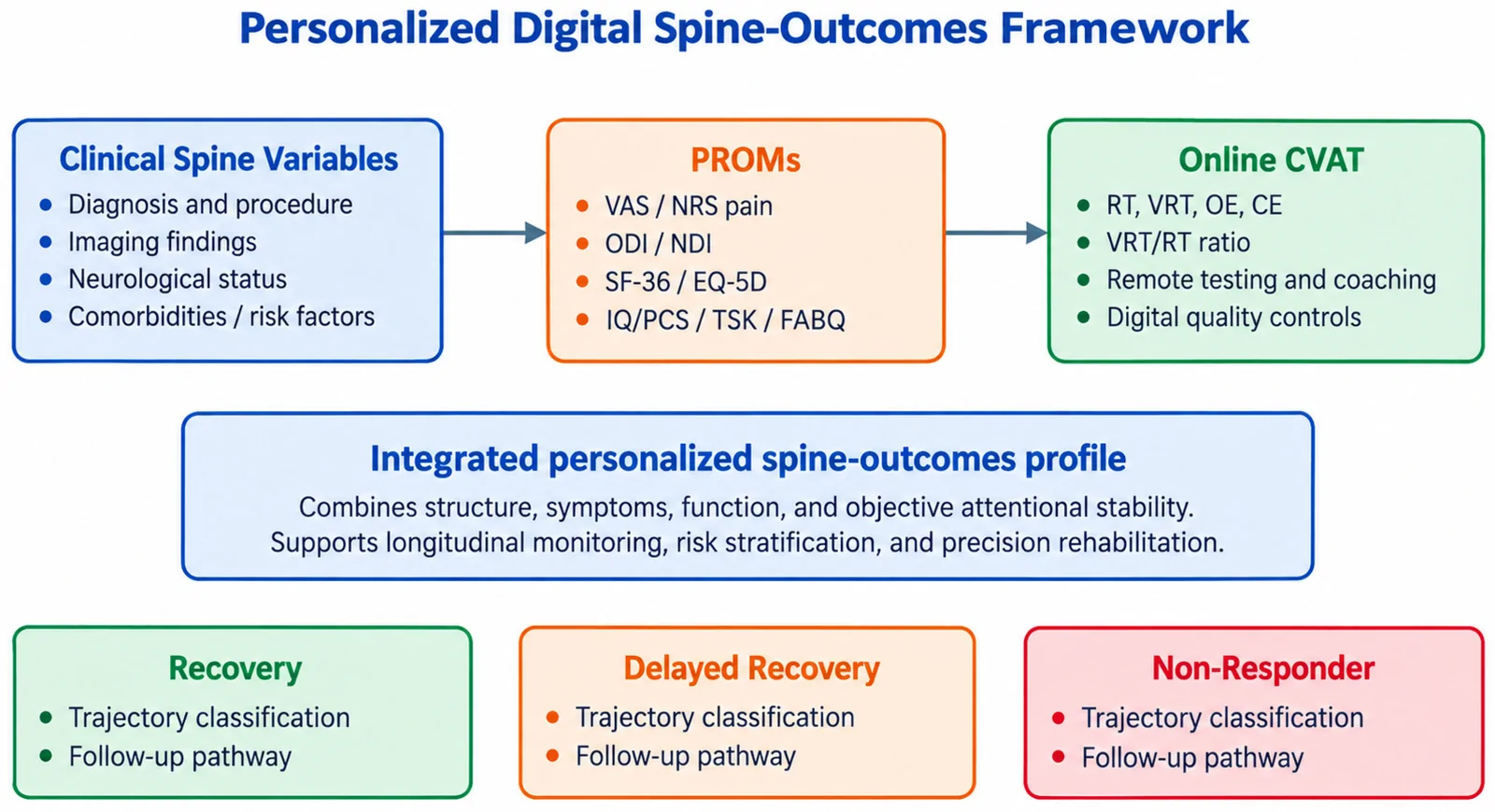
The online 90 s CVAT is integrated with pain scores, PROMs, clinical examination, imaging, surgical variables, medication exposure, and rehabilitation outcomes. The model supports a multidimensional interpretation of recovery in which attentional stability is treated as an adjunct objective performance domain. Integrated recovery and routing phenotypes remain hypotheses for prospective validation, not current clinical decision rules.

**Figure 2:**
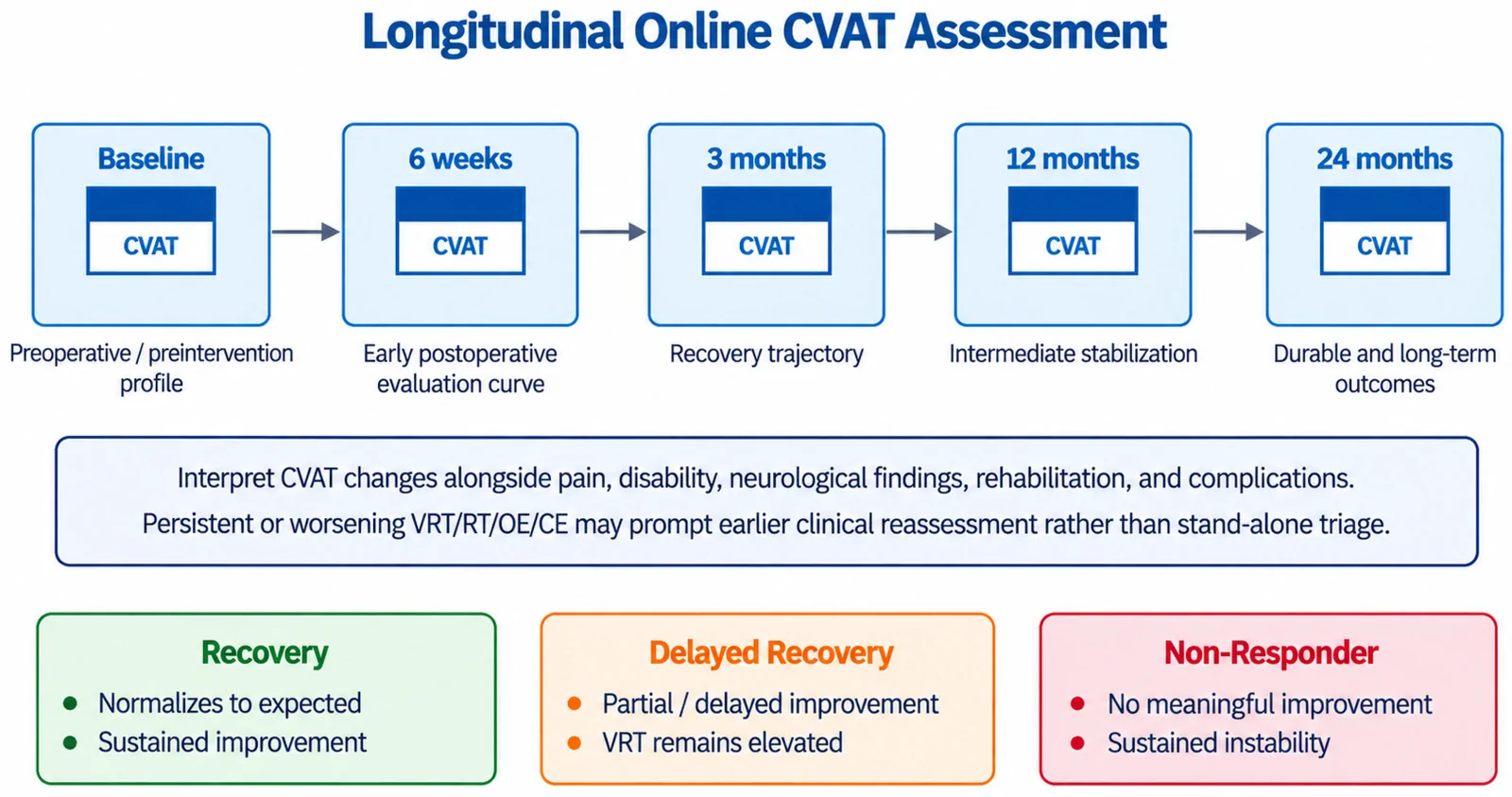
Patients complete baseline online CVAT testing before surgery or intervention, followed by repeat testing at early, intermediate, and long-term follow-up. Serial CVAT results are interpreted alongside pain, disability, neurological status, functional recovery, and complications. The 6-week, 3-month, 12-month, and 24-month intervals are author-proposed pragmatic spine-follow-up anchors: 6 weeks samples early healing and medication change, 3 months the initial recovery trajectory, 12 months durable outcome, and 24 months late durability, recurrence, or reoperation. These are not validated CVAT-specific intervals and may be modified prospectively for an individual disease-specific module.

**Figure 3:**
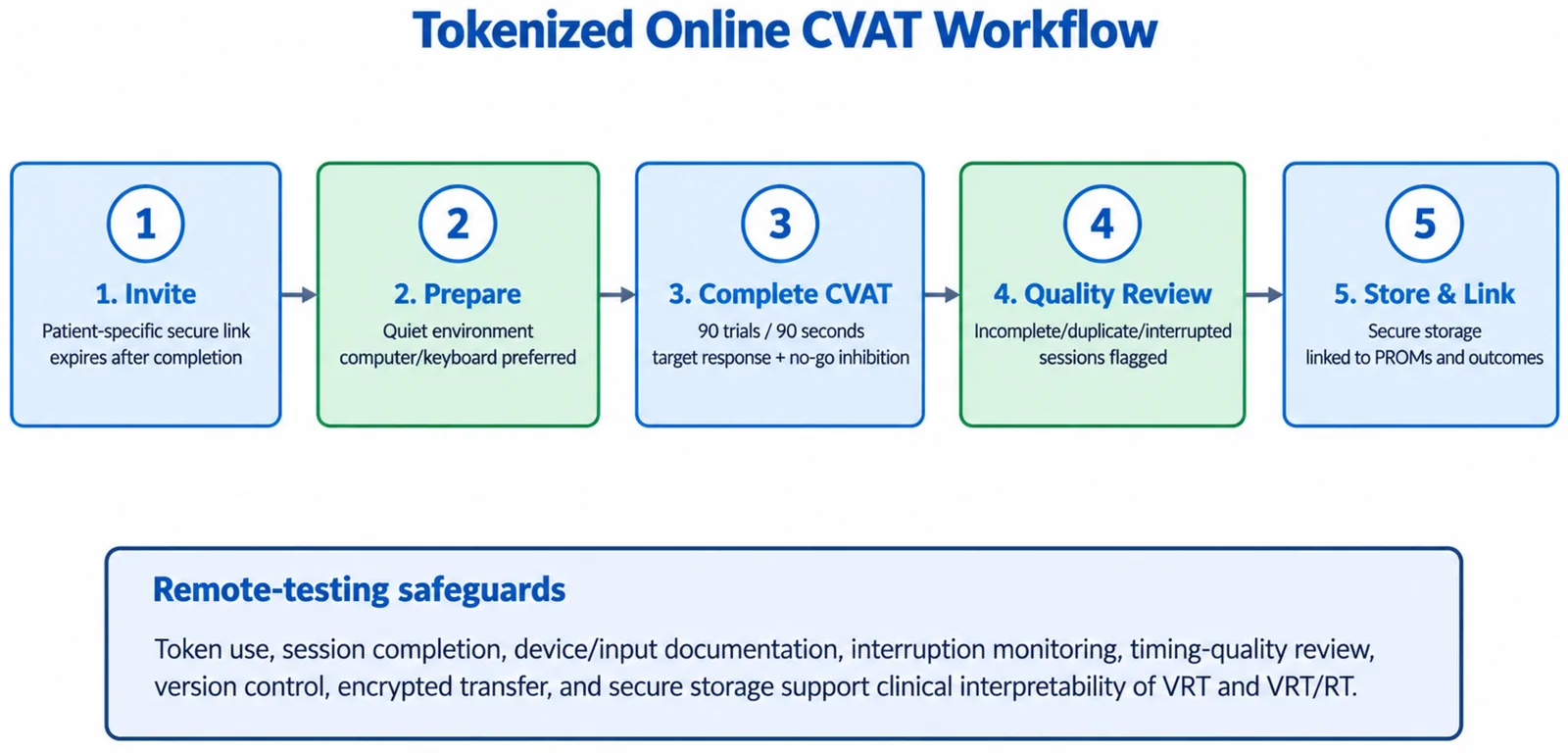
A patient-linked token controls one planned assessment, while standardized preparation, local event logging, scoring, and quality review determine session validity. Tokenization limits duplicate attempts but does not independently authenticate the participant or prevent incomplete sessions; failed, interrupted, and incomplete attempts remain visible in the audit trail.

**Figure 4:**
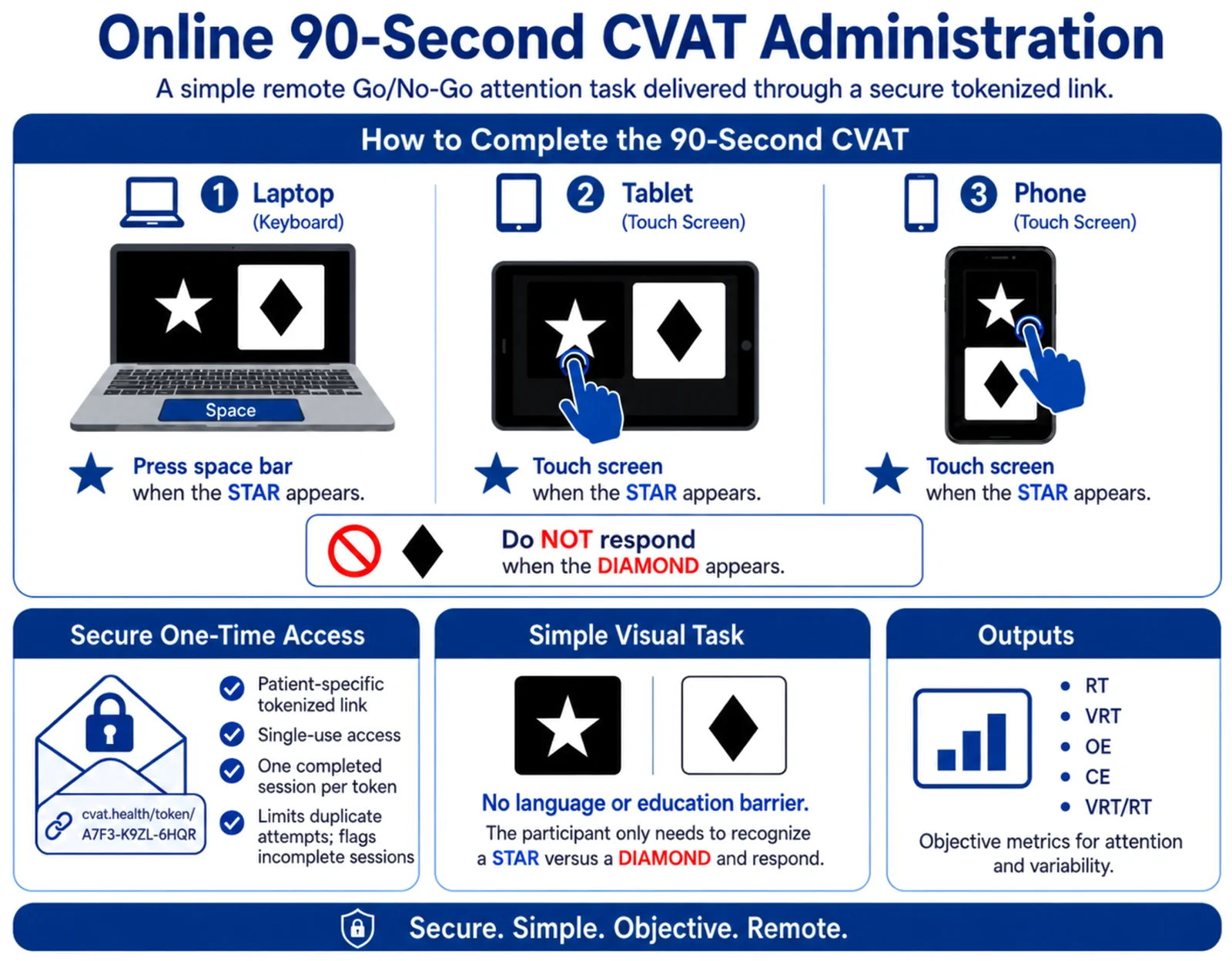
The online Continuous Visual Attention Test (CVAT) can be completed on a laptop, tablet, or phone, although a physical keyboard and the same device/input method across visits are preferred until device equivalence is established. Participants respond to the star stimulus and withhold responses to the diamond stimulus. A patient-specific, single-use tokenized link limits uncontrolled repeat attempts; it does not by itself authenticate identity, prevent link sharing, or guarantee completion. Incomplete, interrupted, or duplicate sessions are retained in the audit trail and flagged. Outputs include reaction time (RT), variability of reaction time (VRT), omission errors (OE), commission errors (CE), and VRT/RT.

**Figure 5:**
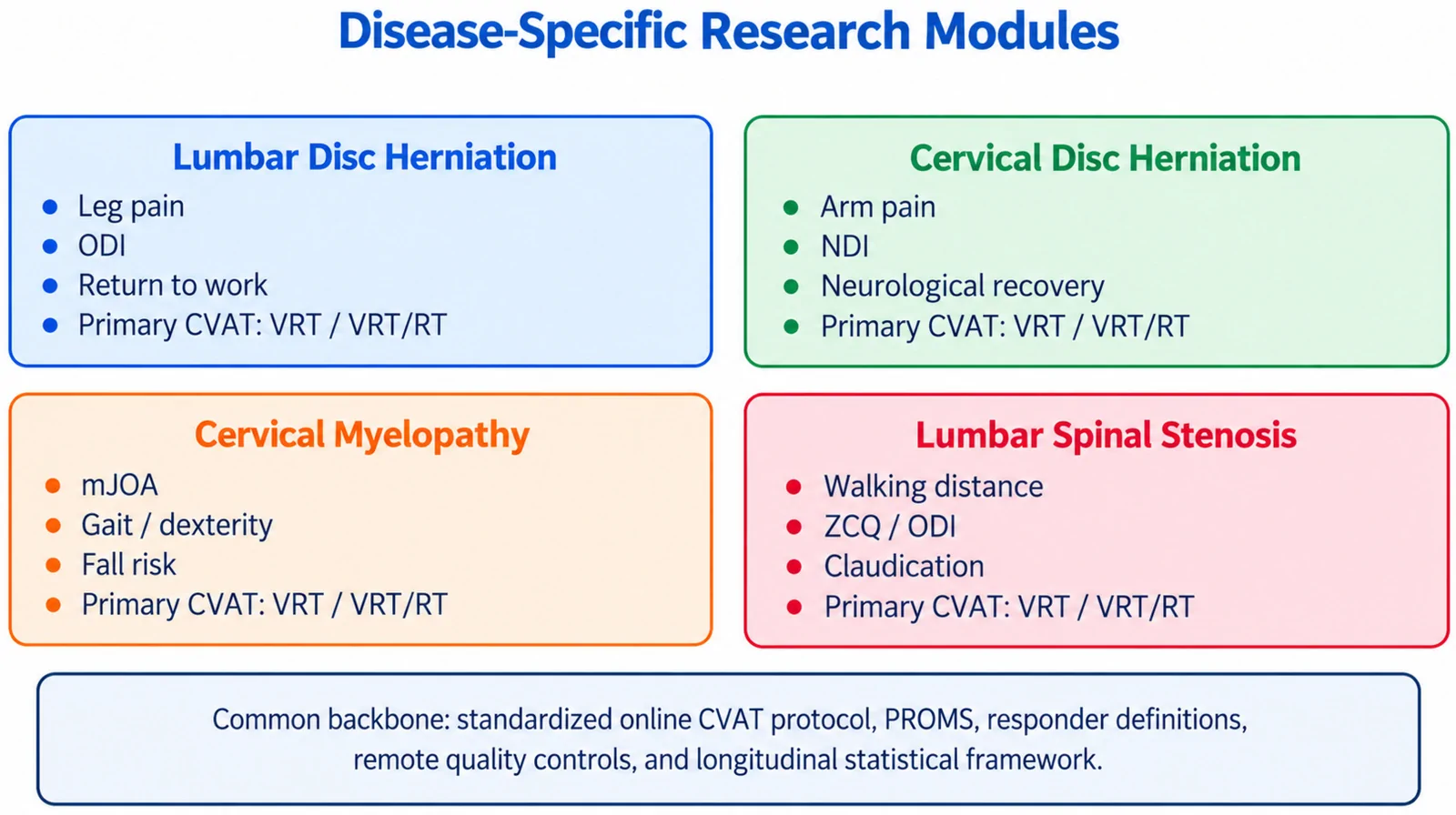
Disease-specific research modules are proposed for lumbar disc herniation/radiculopathy, cervical disc herniation/radiculopathy, cervical myelopathy, and lumbar spinal stenosis. Although each module incorporates diagnosis-specific patient-reported outcome measures (PROMs) and clinical outcomes, all modules use a common online CVAT protocol with standardized task administration, scoring, metadata capture, remote-testing safeguards, and statistical analysis.

**Figure 6:**
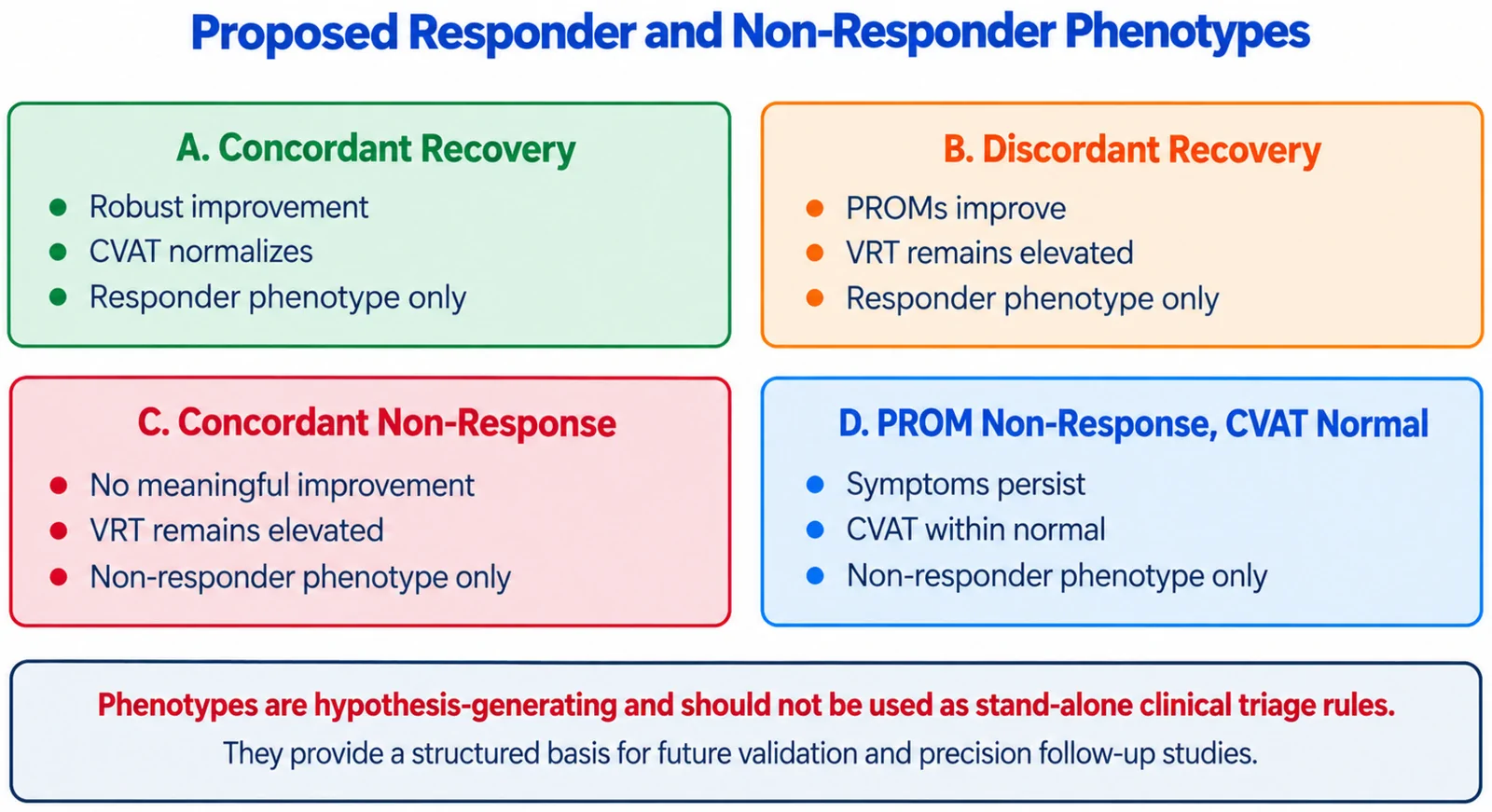
Four exploratory PROM/CVAT phenotypes are proposed for future validation: concordant recovery, discordant recovery, concordant non-response, and PROM non-response with normal CVAT performance. These phenotypes are intended to test whether CVAT provides incremental prognostic information beyond patient-reported outcome measures (PROMs). They are hypothesis-generating constructs rather than validated clinical routing categories and should not be used to trigger medication changes, referral, rehabilitation escalation, or additional diagnostic testing during validation studies.

**Table 1:** Numbered remote-testing and data-quality safeguards for the online 90 s CVAT.

No.	Safeguard	Protocol Requirement	Reason
1	Identity and token	Link the single-use token to a pseudonymous study ID; verify identity separately through the approved portal, a second factor, or staff-confirmed release.	Limits uncontrolled repeat access without treating the token itself as proof of identity or protection against link sharing.
2	Practice	Use a 10 s error-free practice; permit one repeat after an OE or CE; stop after a second failed practice.	Confirms task comprehension and response capability before the scored session.
3	Completion/replacement	Require completion of all 90 trials. Issue one replacement token only after a documented technical failure; retain the original attempt and failure reason.	Prevents selective retesting while allowing recovery from a genuine technical failure.
4	Device/input consistency	Prefer a desktop/laptop with a physical keyboard; keep device, browser family, input method, and response hand constant at follow-up whenever feasible.	Reduces timing heterogeneity and improves interpretability of longitudinal change.
5	Timing/metadata	Use client-side monotonic, high-resolution timestamps; record device, browser, OS, input method, display/refresh data, and task/scoring build.	Makes timing provenance and version-specific sensitivity analyses reproducible.
6	Focus/interruption	Log focus/visibility changes, reloads, network interruptions, timing gaps, and assistance; invalidate sessions with unresolved technical interruption.	Helps distinguish behavioral variability from environmental or software artifacts.
7	Scoring validity	Require at least 37 valid correct-target RTs; flag OE ≥ 50%, CE = 18 with OE = 0, anticipatory responses, and distributional outliers for review.	Applies explicit reproducible criteria while preserving auditable validity and exclusion decisions.
8	Context	Record time of day, sleep, current pain, medication use/timing/change, caffeine, alcohol or recreational drug exposure, acute illness, distraction, assistance, and testing location.	Captures modifiable state and context factors for covariate and sensitivity analyses.
9	Version control	Lock the task/scoring version within a module; prospectively bridge versions before pooling data across software changes.	Prevents software changes from masquerading as clinical change.
10	Security	Use authenticated access, pseudonymization, encryption in transit and at rest, least-privilege access, audit trails, backup, retention/deletion, and breach-response plans.	Protects participants and supports regulatory data integrity.
11	Digital inclusion	Record eligible nonparticipation, device/access barriers, usability, burden, assistance, and technical failures.	Quantifies selection bias in older, disadvantaged, or technology-inexperienced patients [13].

**Table 2:** Established clinical outcomes, proposed adjunct CVAT questions, and future routing hypotheses by spine-study module.

Study Module	Established Clinical Outcomes	Proposed Adjunct CVAT Question	Future Routing Hypothesis (Requires Validation)
Lumbar disk herniation	Leg/back pain, ODI, neurological findings, recurrence, activity/return to work	Do VRT trajectories parallel or add to radicular pain and disability recovery?	A validated early trajectory might refine follow-up intensity for delayed recovery; no current routing rule.
Cervical disk herniation/radiculopathy	Neck/arm pain, NDI, motor/sensory findings, satisfaction	Does attention-related consistency add information beyond symptom and neurological recovery?	Future discordance may identify a need for reassessment only after prospective utility testing.
Cervical myelopathy	mJOA, gait, balance/falls, hand motor function and dexterity	Is VRT associated with outcome after accounting explicitly for response-hand impairment?	Any future monitoring role requires discriminant validation against motor variability.
Lumbar spinal stenosis	Walking/claudication, ODI, ZCQ, quality of life, satisfaction	Do walking and claudication changes parallel VRT after adjustment for age, sleep, medication, and device?	A validated adjunct may refine longitudinal monitoring in older adults; digital exclusion must be quantified [13].
Revision/persistent spinal pain	Pain, ODI/NDI, analgesic burden, revision status, satisfaction, function	Is a VRT trajectory incrementally associated with a prespecified non-response outcome?	A future multidisciplinary routing hypothesis must outperform decisions based on conventional data alone.

**Table 3:** Operational definitions, evidence basis, limitations, and protocol role of CVAT variables.

Variable	Exact Calculation	Published Construct Association	Important Alternatives/Limitations	Protocol Role
RT	Arithmetic mean (ms) of valid correct-target latencies from 100 to <1000 ms.	Intrinsic alertness/processing speed in the CVAT literature.	Motor/visual speed, age, fatigue, pain, medication, device latency.	Secondary; covariate in VRT sensitivity model.
VRT	Sample SD (ms) of the same valid correct-target latencies.	Trial-to-trial consistency; operational sustained-attention indicator [1,2,3].	Mean speed, motor variability, sleep/mood, motivation, device/context; not process-pure.	Primary performance endpoint.
VRT/RT	VRT divided by mean RT; dimensionless intraindividual coefficient of variation.	Relative variability after scaling by mean speed.	Denominator dependence and ratio-distribution problems.	Key secondary/sensitivity; analyze separately from RT and VRT.
OE	Target without a valid response in the 1000 ms trial epoch; count and proportion of 72.	Focused attention/target detection.	Comprehension, distraction, motor inability; floor/ceiling and invalidity at ≥50%.	Secondary count/binomial outcome.
CE	Any response during a non-target trial; count and proportion of 18.	Response inhibition in CVAT literature.	Only 18 opportunities; limited precision, floor/ceiling, spillover/misunderstanding.	Secondary/exploratory count/binomial outcome.

**Table 4:** Prespecified hierarchy of common-core analyses and stage gates.

Stage	Primary Purpose/Estimand	Prespecified Analysis	Status and Gate
Feasibility	Valid scored sessions/initiated assessments at each time point	Proportion and 95% CI; failure reasons; device/access/usability/burden summaries	The completed online cohort yielded 401 analyzable sessions; each module must quantify its own denominators and rates.
Technical reliability	VRT test–retest error and supervised–remote/device agreement when interchangeability is claimed	Absolute-agreement ICC with CI; Bland–Altman bias/limits; sequence and device effects	Required before interpreting small within-person change or claiming cross-mode interchangeability.
Construct/reference	Convergent attention relationship; discrimination from motor/mood; predefined known groups	Correlation/regression models; age/education/device distributions; motor sensitivity	Published online chronic-pain known-groups validity is supported, with additional replication in the independent online cohort; convergent/discriminant validity and norms remain to be extended.
Longitudinal responsiveness	Baseline-adjusted VRT change or time-by-clinical-outcome interaction	Mixed-effects model with participant/site structure and prespecified covariates	Primary clinical estimand selected by each module.
Secondary CVAT outcomes	VRT/RT, RT, OE, CE and alternate time points	Separate ratio/covariate models; count/binomial models; Holm/FDR adjustment [23,24]	Secondary/exploratory; report floor/ceiling.
Incremental prognosis	Added value of CVAT beyond locked clinical/PROM base model	Calibration, discrimination, Brier score, bootstrap optimism correction and external validation	Only with adequate sample/events; association is not prediction.
Clinical utility	Net benefit and impact of a prespecified routing rule	Decision-curve analysis followed by prospective impact study	Only after validated thresholds and external prognosis.

**Table 5:** Author-proposed longitudinal schedule and rationale for prospective spine-study modules.

Time Point	Clinical Data	PROMs	Online CVAT	Protocol Status and Rationale
Baseline/preoperative	Diagnosis, imaging, exam, motor/dexterity, medication, procedure plan	Pain plus diagnosis-specific disability, sleep, mood, cognition/function	Required	Required reference for prospective within-person change.
6 weeks	Complications, medication change, early healing/rehabilitation	Pain, disability, satisfaction, sleep	Recommended	Author-proposed early recovery and medication-context anchor.
3 months	Rehabilitation, work/activity and neurological progress	Pain, disability, function	Required	Default primary early recovery time point; module may justify another.
6 months	Functional plateau or ongoing treatment	Pain, disability, quality of life	Optional	Adds trajectory resolution when clinically justified.
12 months	Durable outcome, complications and revision/intervention status	Pain, disability, quality of life, work/activity	Required	Durable clinical responder assessment.
24 months	Recurrence, reoperation and late durability	Pain, disability, quality of life	Extension	Long-term phenotype; optional if outside the module horizon.

**Table 6:** Staged roadmap from technical validation to CVAT-informed clinical utility.

Stage	Question	Minimum Design	Key Outputs	Gate to Next Stage
0. Analytical/technical	Are stimulus and response times accurate for this build?	Bench timing and event-log verification by version/browser/device	Timestamp error, dropped frames, focus logging, version record	Errors within justified limits.
1. Feasibility	Can representative spine patients complete the workflow?	The completed 401-session online cohort described above plus prospective module-specific denominators, including older and digitally inexperienced patients	Completion, invalidity, failure, burden, acceptability, exclusion bias	Operational deployment demonstrated in the completed dataset; acceptable prespecified module-specific feasibility required.
2. Reliability/equivalence	Is change distinguishable from measurement error, and are modes interchangeable when required?	Paired test–retest and, when relevant, supervised/remote and keyboard/touchscreen/device strata	ICC, SEM/MDC, Bland–Altman, practice and formal equivalence	Adequate reliability; agreement required for any interchangeability claim.
3. Construct/reference	Does VRT behave as an attention-related measure distinct from motor/mood?	Existing published online chronic-pain known-groups evidence plus the independent online replication cohort, comparator attention task, motor/dexterity and mood measures	Convergent/discriminant validity; age/education/device norms	Known-groups signal replicated in published and unpublished datasets; extend construct performance and reference values.
4. Responsiveness	Does CVAT change with recovery beyond measurement error?	Prospective baseline/follow-up cohorts with external anchors	Trajectory, SEM/MDC, anchor/distributional meaningful change	Independent threshold confirmation.
5. Incremental prognosis	Does CVAT improve prediction beyond PROMs and clinical factors?	Adequately powered development plus external validation	Calibration, discrimination, Brier score, clinical strata	Useful externally validated model.
6. Clinical utility	Does CVAT-informed routing improve decisions or outcomes?	Prospective impact/utility study using locked rule	Net benefit, actions, outcomes, burden, harms and equity	Only then consider routine use.

## Data Availability

No new participant-level data were collected or analyzed specifically for this protocol article. Summary findings from the separately prepared 401-participant study are reported in the text as unpublished study data (authors’ data on file) solely to characterize the existing evidence base. The participant-level data underlying that study are governed by its data availability statement, IRB requirements, participant privacy protections, and any applicable data-use agreement. Future modules will state their de-identification, access, code, metadata, and sharing plan in the module-specific registration and ethics materials, subject to participant consent and applicable law.

## Abbreviations

The following abbreviations are used in this manuscript:

**Abbreviation**	**Definition**
CE	Commission errors
CVAT	Continuous Visual Attention Test
ICC	Intraclass correlation coefficient
MCID	Minimum clinically important difference
MDC	Minimum detectable change
mJOA	Modified Japanese Orthopaedic Association score
NDI	Neck Disability Index
NRS	Numeric rating scale
ODI	Oswestry Disability Index
OE	Omission errors
PROMs	Patient-reported outcome measures
RT	Reaction time
SOA	Stimulus-onset asynchrony
VAS	Visual analog scale
VRT	Variability of reaction time
ZCQ	Zurich Claudication Questionnaire
